# Editorial: New insights into renal fibrosis and therapeutic effects of natural products volume II

**DOI:** 10.3389/fphar.2022.1053408

**Published:** 2022-10-28

**Authors:** Dan-Qian Chen, Yan Guo, Zhi-Yong Guo, Yu-Ping Tang

**Affiliations:** ^1^ Key Laboratory of Shaanxi Administration of Traditional Chinese Medicine for TCM Compatibility, Shaanxi University of Chinese Medicine, Xi’an, China; ^2^ Department of Emergency, China-Japan Friendship Hospital, Beijing, China; ^3^ Department of Internal Medicine, University of New Mexico, Albuquerque, NM, United States; ^4^ Department of Nephrology, Changhai Hospital, Naval Medical University, Shanghai, China

**Keywords:** natural product, renal fibrosis, therapeutic target, active component, Chinese herbal medicine

## Introduction

Renal fibrosis which begins as an abnormal tissue regeneration process is the final and common outcome of various chronic kidney diseases (CKD) (Li et al., 2022). Sustained chronic inflammation, myofibroblast activation, epithelial-to-mesenchymal transition (EMT) and extracellular matrix (ECM) accumulation are the major characteristics of renal fibrosis (Chen et al., 2018a). Renin angiotensin aldosterone system is the first-line therapy for CKD in clinics. Although the blockade of renin angiotensin aldosterone system attenuates renal fibrosis, their effects are limited and hardly delay renal fibrosis and CKD progression (Romero et al., 2015). The therapeutic candidate and strategy that specifically target the pathogenesis of fibrosis are urgently needed, which highlights the importance of identifying novel therapeutic targets and mechanisms.

Natural products have been recognized as promising therapeutic candidates for renal fibrosis and CKD treatment, and have yielded favorable efficacy in clinics (Chen et al., 2018b). However, the mechanisms underlying natural products against renal fibrosis are partially unclear which hinders their clinical application. Emerging evidences are beneficial to provide comprehensive acknowledge and guide clinical rational use of natural products.

The present Research Topic aims to collate manuscripts reporting or describing new insights into renal fibrosis and therapeutic effects of natural products. After rigorous peer reviews, a total of 16 manuscripts were published. These manuscripts report the novel mechanisms of renal fibrosis, the therapeutic effects and targets of natural products, and the high-quality evidences from clinical trials.

### New insights into the underlying mechanisms of renal fibrosis

Several novel therapeutic targets and strategies for renal fibrosis in diabetic kidney disease (DKD) are discussed. Liang et al. identified E2F transcription factor 1 (E2F1) as a promising therapeutic target for DKD treatment by acting on the senescence of renal tubular epithelial cells. E2F1 was upregulated in high-glucose-induced renal tubular epithelial cells and DKD animal model. Treatment with metformin suppressed cellular senescence of renal tubular epithelial cells to alleviate renal injury through modulating E2F1. Xiao et al. explored the anti-fibrotic mechanism of bone morphogenetic protein (BMP)-7 in diabetic tubulointerstitial fibrosis. Functionally, BMP-7 alleviated diabetic renal injury by upregulating Id2 protein levels through the BMP-7–MAPK signaling pathway. Further experiments demonstrated that oxymatrine ameliorated EMT process to delay DKD progression by suppressing renal tubulointerstitial fibrosis *via* BMP-7–MAPK pathway. Guo et al. identified insulin-like growth factor-1 receptor (IGF1R) as a novel therapeutic target for DKD treatment *via* modulating EMT process. Treatment with sodium-glucose cotransporter 2 (SGLT2) inhibitor, dapagliflozin, significantly decreased IGF1R levels in plasma samples from patients with DKD and DKD animal model. The suppression of SGLT2/IGF1R/PI3K signaling served as a key mediator in blocking EMT process. Zhang et al. explored the relationship between serum metabolites and gut microbiota in DKD. They found that isomaltose, D-mannose, galactonic acid, citramalic acid, and prostaglandin B2 significantly increased, while 3-(2-hydroxyethyl)-indole, 3-methylindole, and indoleacrylic acid decreased in the DKD model, which involved in the dysfunction of *g_Eubacterium_nodatum_group*, *g_Lactobacillus*, and *g_Faecalibaculum*. These results reveal the potential metabolic and microbial targets for DKD treatment.

Autophagy is traditionally known for its vital role in maintaining homeostasis, structure, and function of the kidney (Chen et al., 2022). Fu et al. found a novel mechanism that persistent autophagy after acute kidney injury (AKI) was detrimental, which induced pro-fibrotic cytokines in renal tubular cells, promoted renal fibrosis and CKD progression. Treatment with autophagy inhibitors substantially blocked repeated low dose cisplatin-induced secretion of pro-fibrotic cytokines in renal tubular cells. In addition, Zhou et al. highlighted the important role of microRNAs in primary membranous nephropathy (MN) progression under PM2.5 exposure. Multiple microRNAs participated in primary MN progression and treatment under PM2.5 exposure through immune system cells by acting on the imbalance of Th17/Treg, indicating Th17/Treg balance/imbalance as new insights of primary MN and its therapeutic value.

### New insights into therapeutic effects of natural products against renal fibrosis

Zhao et al. carried out an integrative network pharmacology-based approach and experimental verification to confirm the anti-fibrotic effect of Jian-Pi-Yi-Shen formula (JPYSF) in kidney. Functionally, JPYSF suppressed EMT process to attenuate renal fibrosis *via* inactivating Wnt3a/β-catenin signaling pathway and enhancing E-cadherin expression in 5/6 nephrectomy-induced renal fibrosis rats. Jia et al. reported the anti-fibrotic effect and underlying mechanism of Tongluo Yishen (TLYS) decoction on renal interstitial fibrosis. TLYS decoction exhibited favorable efficacy in improving renal function, delaying renal interstitial fibrosis progression, and inhibiting pyroptosis in unilateral ureteral obstruction rats. TLYS also attenuated hypoxia-induced NRK-52E cell damage *via* the suppression of the NLRP3-mediated pyroptosis. Shenkang injection (SKI) is a commonly used herbal formula in China, and Wang et al. investigated the underlying mechanism of Shenkang injection against renal fibrosis and CKD. Shenkang injection and its active component rhein suppressed kidney function decline and EMT *via* inhibiting renin angiotensin system activation and the hyperactive Wnt/β-catenin signalling pathway in adenine-induced rats and Ang II-induced HK-2 and NRK-49F cells. Focal segmental glomerulosclerosis (FSGS) accounts for nearly 20% of nephrotic syndrome in children and 40% in adults, with characteristics of fibrosis in glomeruli and interstitium. Tan et al. found that Yi-Shen-Hua-Shi (YSHS) granule prevented nephrotic syndrome progression in clinical and animal models of FSGS. A network pharmacology-based and experimental approaches were used to identify that BMP2/Smad signaling pathway was vital for YSHS granule to attenuate FSGS.

Liu et al. discussed the therapeutic mechanisms of Chinese herbal medicine in attenuating renal interstitial fibrosis including increased ECM, renal tubular epithelial cell phenotype transformation, oxidative stress, renal interstitial fibroblast proliferation, activation, and phenotypic transformation, and the activation of cytokines and inflammatory cells. TGF-β, NF-κB, Wnt/β-Catenin, hedgehog, notch, and MAPK-related signaling pathways are the common intervening targets of Chinese herbal medicine. Wang et al. reviewed the pathophysiological rationale for MN treatments and highlighted its clinical diagnosis by autoantibodies against the phospholipase A2 receptor (PLA2R) and thrombospondin type-1 domain-containing protein 7A (THSD7A) antigens. *Astragalus membranaceus*, *Tripterygium wilfordii*, and Astragaloside IV have exhibited beneficial effects for the treatment of MN clinically. Chen et al. reported the latest progress of quercetin, a natural flavonoid, in treating CKD. Quercetin exhibited protection in both AKI and CKD through anti-hyperglycemic, anti-oxidative effects, autophagy promotion, senolytic mechanisms. Tan et al. concluded the potential mechanisms of Cordyceps against renal fibrosis, and its mechanisms involved in the inhibition of oxidative stress and inflammation, the suppression of apoptosis, the modulation of autophagy, and the reduction of extracellular matrix deposition and fibroblast activation. Clinical trials have confirmed the beneficial efficacy of Cordyceps against CKD, but the low quality and significant heterogeneity of Cordyceps preparations prohibit their extensive use. Further evidences from clinical trials are needed for natural products to boost the application beyond China and Asia.

### New insights into clinical therapeutic effects of natural products against renal fibrosis

Here, two multicenter randomized controlled clinical trials provide favorable evidences for natural products against renal fibrosis. Chen et al. carried out a multicenter randomized controlled clinical trial to confirm the nephroprotection of Chuan Huang Fang (CHF) on CKD patients who suffered AKI. Compared to the reduced glutathione (RG)-treated group, more reductions of Scr, BUN, UA, and better improvement of eGFR were observed in RG + CHF group, and the levels of urinary AKI biomarkers and renal fibrosis biomarkers were lower, highlighting CHF as a promising therapeutic candidate. Persistent inflammation associated with recurrent urinary tract infection (rUTI) is a crucial inducement of inflammation-driven renal fibrosis. A multicenter, randomized, controlled clinical trial from Li et al. reported that Tailin formulation (TLF, a Chinese herbal formulation for rUTI treatment) combined with continuous low-dose antibiotic therapy (CLAT) exhibited more favorable efficacy than CLAT alone in reducing rUTI recurrence, the non-infection-related physical symptoms and tubular fibrosis.

## Conclusion

The Research Topic ‘New Insights into Renal Fibrosis and Therapeutic Effects of Natural Products, Volume II’ have collected worthy studies and contributions on the subject of renal fibrosis, highlighting the promising therapeutic property of natural products in pre-clinical and clinical contexts. We hope that you enjoy and gain from the Research Topic which will surely inspire additional natural products-based research in the future.
